# Synthesis of bacteriophage lytic proteins against *Streptococcus pneumoniae* in the chloroplast of *Chlamydomonas reinhardtii*

**DOI:** 10.1111/pbi.12703

**Published:** 2017-03-07

**Authors:** Laura Stoffels, Henry N. Taunt, Bambos Charalambous, Saul Purton

**Affiliations:** ^1^ Algal Biotechnology Group Institute of Structural and Molecular Biology University College London London UK; ^2^ Research Department of Infection University College London Medical School London UK; ^3^ Present address: Algenuity Eden Laboratory Broadmead Road Stewartby UK

**Keywords:** antimicrobials, *Chlamydomonas reinhardtii*, chloroplast, Pal, Cpl‐1, endolysin

## Abstract

There is a pressing need to develop novel antibacterial agents given the widespread antibiotic resistance among pathogenic bacteria and the low specificity of the drugs available. Endolysins are antibacterial proteins that are produced by bacteriophage‐infected cells to digest the bacterial cell wall for phage progeny release at the end of the lytic cycle. These highly efficient enzymes show a considerable degree of specificity for the target bacterium of the phage. Furthermore, the emergence of resistance against endolysins appears to be rare as the enzymes have evolved to target molecules in the cell wall that are essential for bacterial viability. Taken together, these factors make recombinant endolysins promising novel antibacterial agents. The chloroplast of the green unicellular alga *Chlamydomonas reinhardtii* represents an attractive platform for production of therapeutic proteins in general, not least due to the availability of established techniques for foreign gene expression, a lack of endotoxins or potentially infectious agents in the algal host, and low cost of cultivation. The chloroplast is particularly well suited to the production of endolysins as it mimics the native bacterial expression environment of these proteins while being devoid of their cell wall target. In this study, the endolysins Cpl‐1 and Pal, specific to the major human pathogen *Streptococcus pneumoniae,* were produced in the *C. reinhardtii* chloroplast. The antibacterial activity of cell lysates and the isolated endolysins was demonstrated against different serotypes of *S. pneumoniae*, including clinical isolates and total recombinant protein yield was quantified at ~1.3 mg/g algal dry weight.

## Introduction

The increase of antibiotic resistance in the last few decades has severely reduced the reliability of antibiotic treatments (Levy, 2005). In particular, the prevalence and range of multidrug resistant strains has increased alarmingly (Levy and Marshall, 2004; WHO report 2014), and there has been a simultaneous decline in the development of new antibiotics, mainly owing to the lower profitability of antibiotics compared to other drugs (Spellberg *et al*., 2008; Wright, 2012). Health agencies, including the World Health Organization (WHO) and the Infectious Disease Society of America (IDSA), have warned that antimicrobial resistance is an ‘increasingly serious threat to global public health’ and that there is a real risk of falling back into a pre‐antibiotic era in the 21st century (Infectious Diseases Society of America (IDSA) 2011; WHO report 2014). Furthermore, many antibiotics are broad spectrum and therefore each treatment affects the human commensal bacterial flora in addition to the target pathogen. Not only does this increase the patient's susceptibility to opportunistic bacterial and fungal pathogens, there is also increasing evidence that disruption to the natural microbiota can play a key role in conditions such as obesity, type 1 diabetes, inflammatory bowel disease, allergies and asthma (Blaser, 2011; Chen and Blaser, 2007). Repeated exposure to broad spectrum antibiotics can also result in the spread of antimicrobial resistance genes within the human microbiome, which in turn can be passed on to pathogenic bacteria (Schmelcher *et al*., 2012). With the availability of enhanced diagnostic methods it would therefore be highly desirable to develop narrow spectrum antibiotics that specifically target the pathogen, thus reducing the collateral effects on the commensal bacterial flora and in turn the spread of antimicrobial resistance genes (Blaser, 2011).

Endolysins are bacteriophage‐encoded enzymes that accumulate in the cytoplasm of infected bacterial cells throughout the lytic cycle (Loessner, 2005). Towards the end of this cycle, the next generation of phage particles is assembled, and the synthesis of a second phage protein known as a holin is initiated. The holin creates pores in the plasma membrane allowing the endolysin to exit the cytoplasm and attack the cell wall of the infected bacterium (Schmelcher *et al*., 2012; Young *et al*., 2000). Cleavage of specific bonds within the peptidoglycan results in the lysis of the host cell and the release of the phage progeny (Loessner, 2005). In the case of Gram‐positive bacteria, where the peptidoglycan layer is exposed to the bulk solution, such enzymes have been shown to function as ‘exo‐lysins’, lysing the target bacterium when applied externally (Schmelcher *et al*., 2012). Gram‐positive targeting endolysins are also typically highly specific for the cell wall of the bacterial species from which they are naturally produced (Borysowski *et al*., 2006), which allows for their use as targeted antibacterial treatments that specifically eliminate the pathogen without harming the commensal human microbiota, in contrast to many conventional antibiotics.

Bacteriophages and their host bacterial strains have co‐evolved over billions of years, with the functionality of the phage endolysin representing a key factor in the ensuing arms race: if a host strain develops effective resistance against endolytic attack, then the phage progeny will not be released and that particular phage lineage will not continue. It is thus inferred that any modern‐day phage must necessarily have evolved to target components in the cell wall that are essential for bacterial viability, and as such are highly immutable. It was proposed that the development of bacterial resistance against an endolysin‐based antibiotic would be less frequent than that seen for conventional antibiotics (Schmelcher *et al*., 2012), and indeed attempts to induce resistance by treating bacterial populations to sublethal doses of endolysin over several generations (even in combination with a mutagen) have failed to generate any observable effect (Loeffler *et al*., 2001; Schuch *et al*., 2002). Recombinant endolysins have been successfully tested against several different pathogenic bacteria *in vitro* and in a range of animal models (Entenza *et al*., 2005; Loeffler *et al*., 2001; Schuch *et al*., 2002). The high activity, specificity and low occurrence of resistance make endolysins highly promising candidates for use as novel antibacterial agents in human and veterinary medicine.

Despite the benefits associated with endolysins, they are yet to achieve clinical adoption. The reason for this is largely considered to be economic; the production costs for recombinant therapeutic proteins are significantly higher than small molecule bioactives such as traditional antibiotics (Dove, 2002). It is therefore desirable to develop an inexpensive production system for recombinant endolysins that also fulfils all safety criteria for the production of therapeutic proteins. Microalgae offer several potential advantages for the production of recombinant proteins over more established expression platforms, including a low cost of cultivation using media with simple nutrient requirements and the ability to rapidly develop and scale‐up production. A number of key species have also been granted GRAS (Generally Recognised as Safe) status for human consumption – as such, the removal of any endogenous toxins and infectious agents from the product is not a concern (Dove, 2002; Rasala *et al*., 2010; Specht *et al*., 2010). Furthermore, the ability of photosynthetic organisms to grow using just light, inorganic substances and CO_2_ has the potential to decrease the overall carbon footprint of the production process. Finally, microalgae can be grown in full containment under sterile conditions in simple photobioreactors (PBRs) (Gimpel *et al*., 2014; Specht *et al*., 2010), significantly reducing the potential for release of transgenes to the environment and reducing the risk of environmental contamination of the production system.

The eukaryotic microalga *Chlamydomonas reinhardtii* offers all the aforementioned advantages together with established techniques for the expression of foreign genes from the chloroplast and nuclear genomes (Almaraz‐Delgado *et al*., 2014; Fuhrmann *et al*., 1999). Expression in the chloroplast is more attractive as the integration of transgenes into the genome occurs via homologous recombination and so the insertion site can be easily defined. In addition, the levels of recombinant proteins that can be achieved in the organelle are markedly higher compared to expression from the nuclear genome (Potvin and Zhang, 2010). Furthermore, the chloroplast compartment offers several specific advantages for the production of endolysins given its evolution from a cyanobacterial endosymbiotic ancestor and hence its similarity to the bacterial cells in which endolysins naturally accumulate. For example, (i) recombinant proteins are retained and accumulate exclusively within the chloroplast (Tran *et al*., 2013), but the chloroplast lacks a peptidoglycan cell wall to which the endolysin might bind or cleave during cell breakage, thereby hindering cultivation and purification; (ii) endolysins are likely to have a long‐half‐life in this environment as the endogenous proteases in the chloroplast are homologues of the bacterial proteases to which endolysins have evolved to be resistant (Adam *et al*., 2006); (iii) proteins synthesized in the chloroplast are not subject to post‐translational modifications such as glycosylation, unlike cytoplasmic production platforms. Indeed, the potential exploitation of chloroplasts of plants and microalgae as platforms for the production of therapeutic proteins such as antimicrobials and vaccines has been highlighted in several recent reviews (Bock, 2015; Bock and Warzecha, 2010; Daniell *et al*., 2016; Rasala and Mayfield, 2015).

The biosynthesis of active endolysins in the chloroplast of tobacco was demonstrated by Oey *et al*. (2009a) who showed that the PlyGBS endolysin, which targets *Streptococcus* pathogens, can accumulate in the leaves to levels as high as 70% of total soluble protein. A second study by the same group similarly showed high level production in tobacco of two further *Streptococcus* endolysins, Pal and Cpl‐1 (Oey *et al*., 2009b). Additionally, this study highlighted the limitations of trying to over‐express endolysin genes using an *E. coli* platform, as both of Pal and Cpl‐1 had a lethal effect on the bacterium, potentially caused by unspecific peptidoglycan cleavage at high concentrations or by binding to the cell wall (Oey *et al*., 2009b). The tobacco chloroplast has also been employed to make other types of antimicrobial proteins and peptides that are technically difficult to produce in established platforms. For example, the clinically relevant antimicrobial peptides retrocyclin and protegrin have complex secondary structures with multiple disulphide bonds, but are able to assemble into a functional form in the chloroplast (Lee *et al*., 2011). However, there are major challenges associated with the commercial production of recombinant pharmaceuticals in plants grown in fields or greenhouses, as compared to single cells cultured in contained, sterile fermenter‐based systems. These challenges include both compliance with good manufacturing practice (GMP) regulations, and the potentially high cost of purifying product from plant biomass under these GMP regulations (Fischer *et al*., 2012).

In this study, we have investigated the production of endolysins in the chloroplast of *C. reinhardtii* to examine the suitability of this microalga as a production platform. We have focussed on the Cpl‐1 and Pal endolysins which are specific to the human pathogen *Streptococcus pneumoniae*, as previous studies have demonstrated the antibacterial efficacy of these endolysins both *in vitro* and *in vivo* (Garcia *et al*., 1983; Grandgirard *et al*., 2008; Jado *et al*., 2003; Loeffler *et al*., 2001). We report the generation of transgenic *C. reinhardtii* lines expressing codon‐optimized genes for Pal and Cpl‐1, and the stable accumulation of the endolysins to a level of ~1% of total soluble protein (TSP). Both crude cell lysates and the isolated endolysins show antibacterial activity against *S. pneumoniae*, including clinical isolates with resistance against penicillin and co‐trimoxazole.

## Results

### Creation of marker‐less transgenic lines of *Chlamydomonas reinhardtii* expressing *pal* and *cpl‐1* in the chloroplast

**Figure 1:**
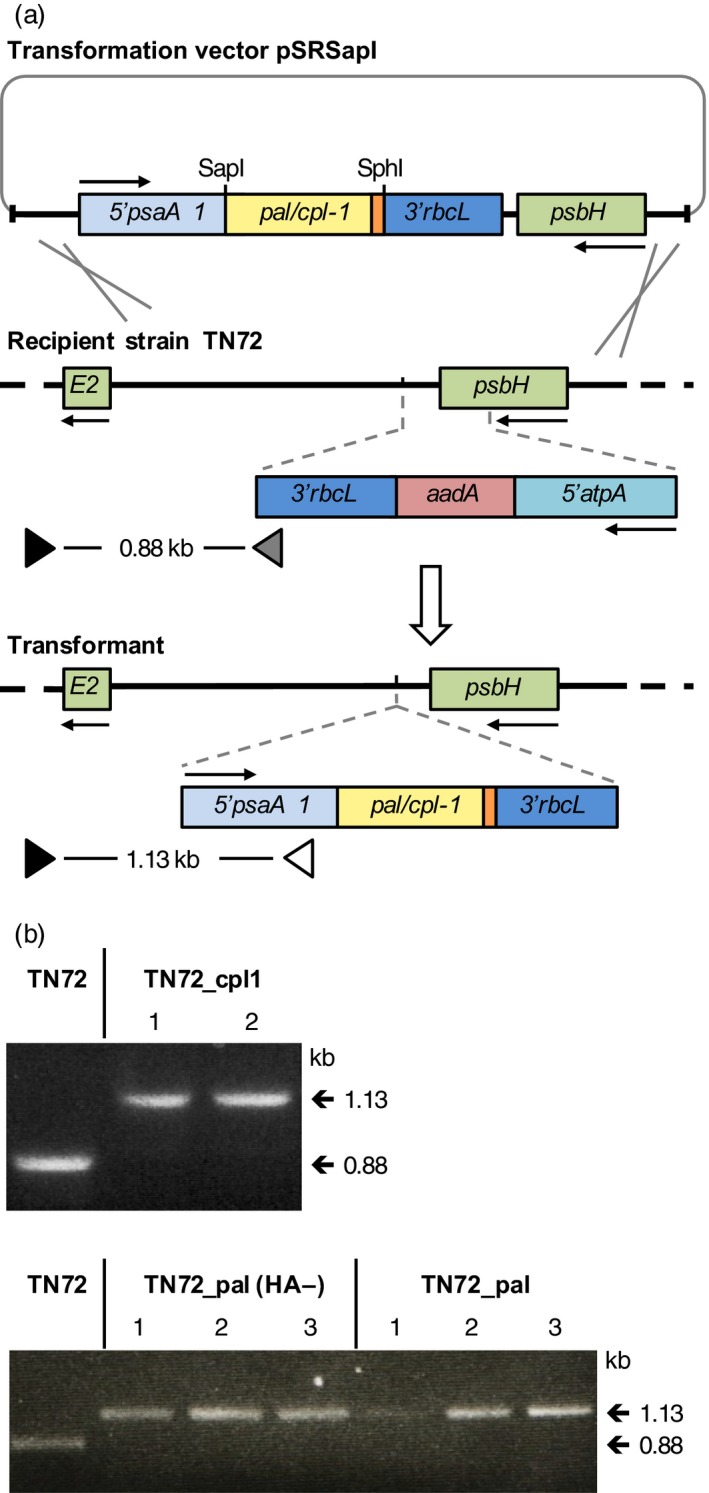
Schematic diagram of the transformation system used for the introduction of *cpl‐1* or *pal* into the chloroplast genome. (a) Homologous recombination (grey crosses) between chloroplast sequences (thick black lines) on the transformation vector pSRSapI and the genome of the recipient strain TN72 allows the introduction of the gene (under the control of the *psaA* exon 1 promoter/5′UTR and *rbcL* 3′ UTR from *C. reinhardtii*) into a neutral locus between *psbH* and *trnE2*. Recombination also replaces the *aadA*‐disrupted copy of *psbH* with a functional copy of this essential photosystem II gene, allowing transformants to be selected by restoration of phototrophic growth (Wannathong *et al*., 2016). (b) PCR screening of putative transformants for correct insertion of *cpl‐*1*, pal(HA‐)* and *pal* and homoplasmicity of the polyploidy chloroplast genome using a set of three primers. Primer F1 (black triangle in (a)) binds to the genome outside of the recombination region. Primer R1 (grey triangle) binds within the *aadA* cassette of TN72 and results together with primer F1 in a product of 0.88 kb. Primer R3 (white triangle) binds within the gene cassette of the transformants and results together with primer F1 in a product of 1.13 kb. Homoplasmy is scored by a band at 1.13 kb and an absence of a 0.88 kb band arising from untransformed copies of the plastome (Wannathong *et al*., 2016).

To investigate the synthesis of endolysins in *C. reinhardtii*, we created transgenic lines in which a synthetic gene encoding either Pal or Cpl‐1 was introduced into the chloroplast genome. The coding regions of the genes were designed based on the codon preference for *C. reinhardtii* chloroplast genes and synthesized *de novo*. (Nakamura *et al*., 2000) (Figure S1). The coding sequence for a C‐terminal human influenza haemagglutinin (HA) epitope tag was also added to each gene to facilitate the detection of the proteins. These synthetic genes are referred to as *pal* and *cpl‐1*. A *pal* gene without the HA‐tag sequence (referred to as *pal(HA‐)*) was also included to investigate whether the HA‐tag has an influence on the stability or activity of the endolysin. As shown in Figure 1a, the genes were cloned into the chloroplast transformation vector pSRSapI that uses the chloroplast *psaA‐1* promoter and 5′ untranslated region (UTR) to drive transgene expression (Young and Purton, 2014). To compare the efficiency of expression, *pal* and *cpl‐1* were also cloned into a second vector, pASapI, that differs from pSRSapI in that it uses the promoter/5′UTR from the *atpA* chloroplast gene (Economou *et al*., 2014). Each vector is designed to target the transgene cassette to a neutral site in the chloroplast genome downstream of the essential photosystem II gene *psbH*, with restoration of phototrophy in the Δ*psbH* recipient line TN72 used for selection of transformants (Young and Purton, 2014). Such transformants therefore lack any antibiotic‐resistance marker and carry the gene of interest (GOI) as the only section of foreign DNA (Figure 1a).

We analysed colonies of putative *C. reinhardtii* transformants for the correct insertion of the endolysin genes into the chloroplast genome and homoplasmicity of the polyploid genome using a three‐primer PCR assay (Economou *et al*., 2014). As shown in Figure 1b, homoplasmic transformants were obtained for pSRSapI_cpl‐1, _pal and _pal(HA‐). Homoplasmic transformants were also obtained for the pASapI_pal and pASapI_cpl‐1 constructs, and also for a control transformant line (TN72_control) lacking the GOI in which TN72 was transformed with the empty pSRSapI vector (data not shown). Furthermore, the correct insertion of the expression cassettes carrying *pal* and *cpl‐1* into the lines TN72_pal and Tn72_cpl‐1 was confirmed by Southern blot analysis using probes binding within the *pal* and *cpl‐1* genes, as well as a probe that binds adjacent to the insertion site of the expression cassette (Figure S2).

### Accumulation of Pal and Cpl‐1 in the *C. reinhardtii* chloroplast

**Figure 2:**
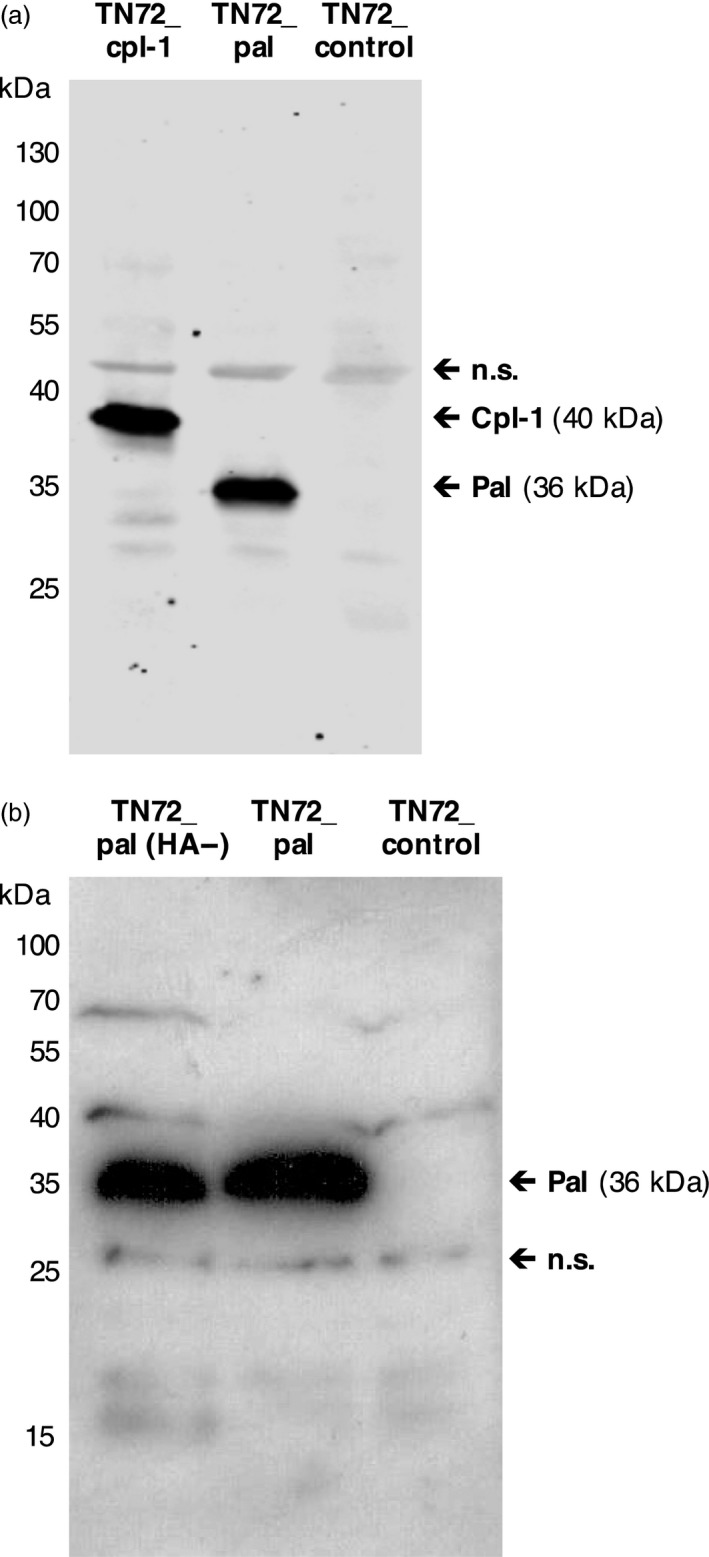
Western blot analysis of endolysin accumulation in transgenic lines. (a) Detection of HA‐tagged Cpl‐1 and HA‐tagged Pal in equivalent loadings of cell extracts from two representative transgenic lines (TN72_cpl1 and TN72_pal) using anti‐HA antibodies, together with the negative control transformant (TN72_control). (b) Comparison of levels of untagged Pal and HA‐tagged Pal in extracts from two representative lines (TN72_pal(HA‐)) using custom made anti‐Pal antibodies. Binding of the different primary antibodies to nonspecific (n.s.) bands within the extracts serves as a loading control.

We analysed representative TN72_cpl‐1 and TN72_pal lines generated using the pSRSapI constructs for the presence of the recombinant protein. This was carried out on crude cell extracts by Western blot analysis using antibodies against the HA‐tag. The Cpl‐1 protein has an expected size of 40 kDa and Pal an expected size of 36 kDa, and as seen in Figure 2a, both proteins are readily detected in the extracts, confirming the accumulation of Pal and Cpl‐1 in the respective lines. For the detection of untagged Pal, antibodies to Pal were raised in rabbits using two synthetic peptides derived from the protein sequence (Figure S3) and used to compare Pal levels in the TN72_pal and TN72_pal(HA‐) lines. As seen in Figure 2b, a 36‐kDa band of similar intensity is seen for both the HA‐tagged and untagged protein indicating that the HA‐tag does not interfere with the stability of this endolysin. Furthermore, we did not observe any reduction in growth rate or biomass production of the Pal and Cpl‐1 producing strains in comparison with TN72_control, indicating that the production of the endolysins in the chloroplast is not detrimental to *C. reinhardtii* growth (shown for TN72_pal in Figure S4).

When we compared the levels of Pal and Cpl‐1 synthesized using the *atpA* promoter/5′UTR with those from the *psaA‐1* promoter/5′UTR, a higher level was seen for both proteins when using the *psaA‐1* elements (Figures S5 and S6), as has been reported previously (Michelet *et al*., 2011). We therefore used the transgenic lines with the *psaA‐1* promoter/5′UTR for all further studies.

**Figure 3:**
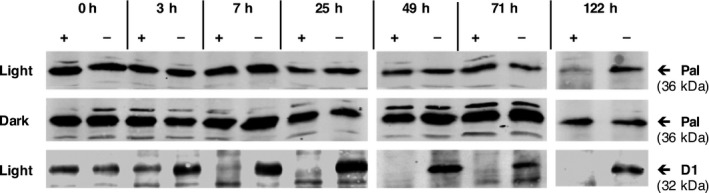
Stability of Pal after inhibition of chloroplast protein synthesis by chloramphenicol. Cultures of TN72_pal were grown under standard conditions to an OD_750 nm_ of 1 before treating half with 500 μg/mL chloramphenicol and incubating for a further 122 h. The experiment was performed both in the light and the dark with samples taken at different time points. Samples were normalized to the same cell density and analysed by Western blotting using anti‐HA and anti‐D1 antibodies. + = Chloramphenicol‐treated cultures, – = untreated controls.

To investigate the stability of the endolysins in the *C. reinhardtii* chloroplast, we grew cultures of TN72_pal to mid‐log phase and specifically inhibited further protein synthesis in the chloroplast with the addition of chloramphenicol. As shown in Figure 3, there is very little decrease in the amount of Pal in the chloramphenicol‐treated cultures compared to the untreated controls even after 71 h, and the protein was still detectable 122 h after the start of the inhibition. In contrast, the endogenous D1 protein (a component of photosystem II) decreased to undetectable levels 7 h after the start of the inhibition. In addition, we found that the stability of Pal was further increased during cultivation in the dark (Figure 3). This indicates a high stability of Pal in the *C. reinhardtii* chloroplast, as has been reported previously for both Pal and Cpl‐1 when produced in the tobacco chloroplast (Oey *et al*., 2009b).

### Isolation and quantification of Pal and Cpl‐1

**Figure 4:**
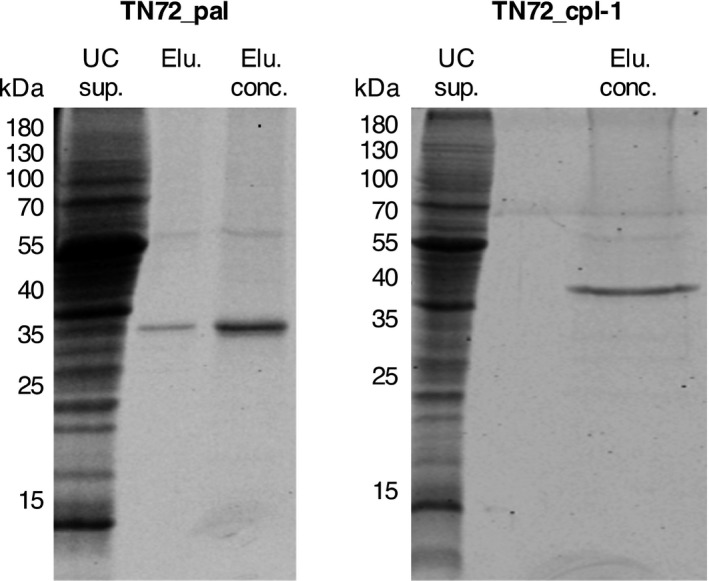
SDS‐PAGE analysis of isolated Pal and Cpl‐1. The recombinant proteins were isolated from the crude cell lysate following ultracentrifugation (=UC sup.) using diethylaminoethanol (DEAE) cellulose with choline as the specific eluent. The elution fractions with the highest amount of Pal and Cpl‐1 were pooled (=Elu.) and concentrated using ammonium sulphate precipitation (=Elu. conc.). The figure shows Coomassie stained gels recorded using the Odyssey^®^ Infrared Imaging system.

For the development of the *C. reinhardtii* chloroplast as a production platform for therapeutic endolysins, it is important to show that the proteins can be extracted and purified in a biologically active state. Furthermore, it was necessary to produce pure standards for the quantification of Pal and Cpl‐1 levels within the algal cell. We isolated the endolysins from crude cell extracts as described in the methods, using ultracentrifugation followed by chromatography with the weak anion exchanger diethylaminoethyl (DEAE) cellulose and choline as specific eluent. Pal and Cpl‐1 bind to choline in the *S. pneumoniae* cell wall, and DEAE is a choline analogue which is commonly used for the affinity chromatography of choline binding proteins (Jado *et al*., 2003; Sanz *et al*., 1988). As the final step, we concentrated Pal and Cpl‐1 by either ammonium sulphate precipitation or with centrifugal concentrators. As seen in Figure 4, the Pal and Cpl‐1 isolated from the algal biomass using this procedure are in a near purified state with only trace amounts of other proteins, including a 55‐kDa protein which is most likely the large subunit of the highly abundant Rubisco enzyme.

To optimize the yield of recombinant endolysin, we first analysed the level of Pal in the algal cell at different growth stages and conditions. This indicated that under mixotrophic growth conditions, the concentration of Pal on a per cell basis is highest during the logarithmic phase and then declines once the cells enter stationary phase (Figures S5, S7 and S8). However, different experiments suggested that a shift from light to dark (*i.e*. heterotrophic conditions) can limit this decline (Figures S7 and 3), possibly due to the inactivity of chloroplast proteases that are dependent on ATP generated through photo‐phosphorylation as reported by Preiss *et al*. (2001). However, heterotrophic cultivation of the transformant line from the start of inoculation resulted in an overall reduction in Pal yield per culture volume because of a reduction in cell density under these conditions when compared to mixotrophic growth (Figure S8). Hence, the maximal yield per culture volume was achieved towards the end of the logarithmic phase under conditions of mixotrophic growth.

**Figure 5:**
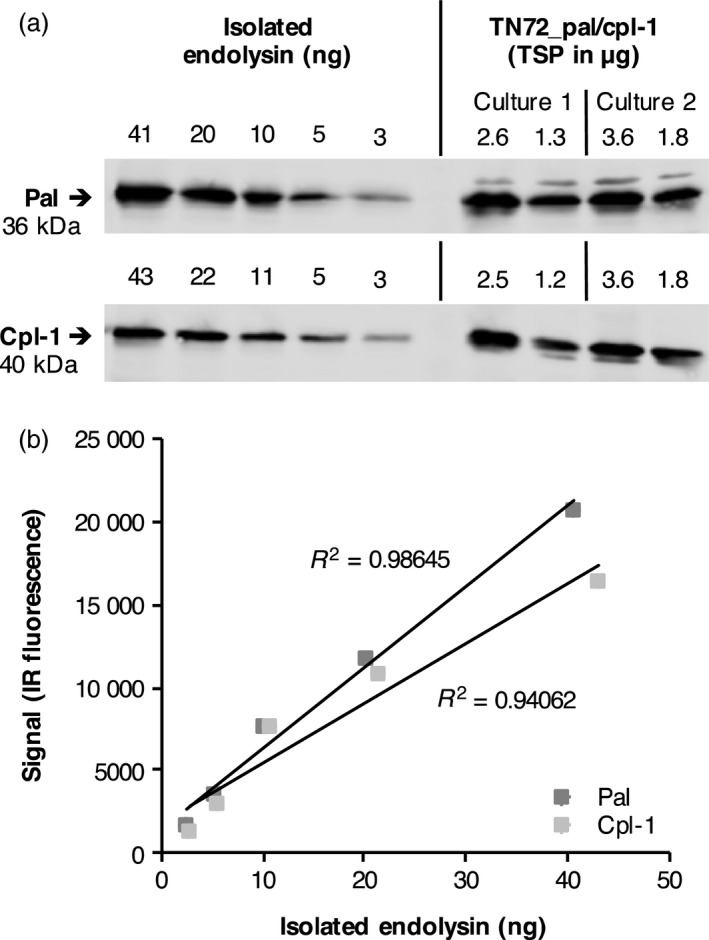
Quantification of Pal and Cpl‐1 accumulation in *C. reinhardtii*. (a) Cell lysates of TN72_pal and TN72_cpl‐1 were compared to a dilution series of known amounts of isolated Pal or Cpl‐1 in Western blot analysis using anti‐HA antibodies and IRDye^®^ secondary antibodies. The amount of total soluble protein (TSP) in the cell lysates is stated above each lane. For each strain, ‘Culture 1’ was grown to an OD_750_ of 2.5 and ‘Culture 2’ to an OD_750_ of 3.0. The cultures were concentrated five times (lane 1) or 2.5 times (lane 2) in comparison with the initial culture volume before preparation of the cell lysates. (b) The IR fluorescence signals of the serial dilutions were plotted against the protein concentration of the isolated endolysin preparations (taking impurities in the isolated proteins into account) and the equations of the resulting trend lines used to calculate the concentration of Pal/Cpl‐1 in the cell lysates.

To quantitate the amount of recombinant protein, we performed Western blot analyses with a dilution series of the isolated endolysins alongside crude cell lysates of TN72_pal and TN72_cpl‐1 taken from mid‐ and late‐logarithmic phase cultures (Figure 5a). Using the Odyssey^®^ imaging system (LI‐COR) to quantify the amount of antigen in the samples (Figure 5b), we found that both Pal and Cpl‐1 represent ~1% of total soluble protein (TSP) in *C. reinhardtii*. This equates to ~1 mg of recombinant protein per litre of culture volume at the end of the logarithmic phase during mixotrophic cultivation, or ~1.3 mg per gram of algal dry weight (Table 1).

**Table 1:** Quantification of Pal and Cpl‐1 accumulation in *C. reinhardtii*

	% of total soluble protein (TSP)	mg endolysin/g of cell dry weight	mg endolysin/L culture volume
Pal	0.9–1.2%	1.3 ± 0.4	1.2 ± 0.4 (Culture OD_750 nm_ of 3.8)
Cpl‐1	1.1–1.2%	1.2 ± 0.7	0.9 ± 0.3 (Culture OD_750 nm_ of 3.0)

### Antibacterial activity of Pal and Cpl‐1

**Figure 6:**
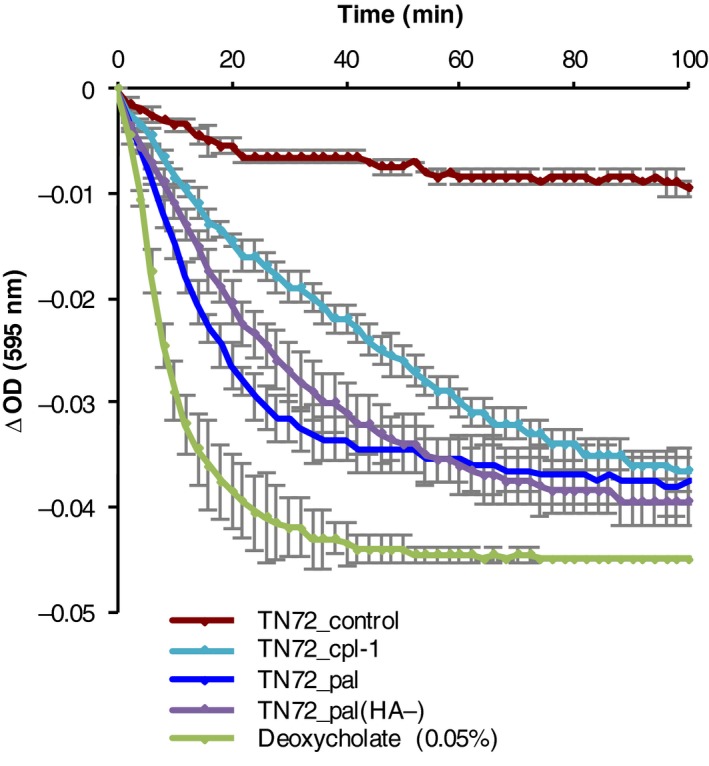
Turbidity reduction assay showing the lytic activity of crude cell extracts containing Pal (HA‐tagged and untagged) and Cpl‐1. *Streptococcus pneumoniae* (16 NP3, serotype 19F) cells were resuspended in Na‐Pi buffer to an OD_595_ of 0.1. Crude extracts of equal concentration of TN72_pal*,* TN72_pal(HA‐)*,* TN72*_*cpl‐1 and TN72_control were added to the *S. pneumoniae* suspension, and the cell lysis was measured as a decrease in OD_595_. Lysis of the bacterium by 0.05% deoxycholate was used as a positive control. Error bars show ± one standard deviation (*n* = 2).

**Figure 7:**
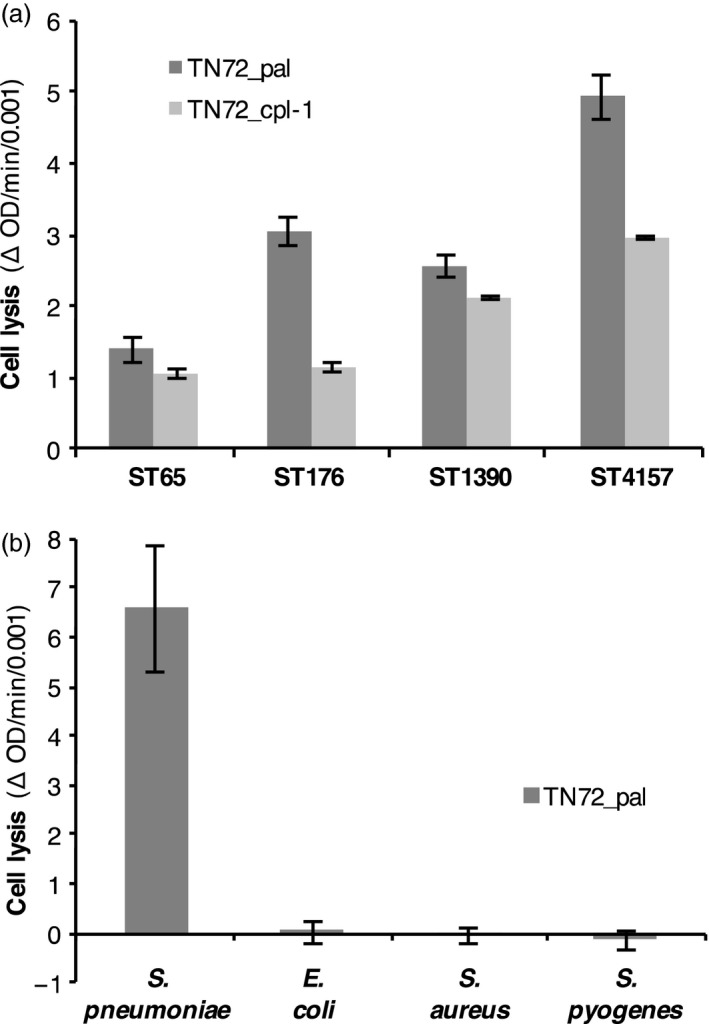
Lytic activity of Pal and Cpl‐1 against clinical isolates of *S. pneumoniae* and specificity of Pal for *S*. *pneumoniae*. (a) Suspensions of clinical isolates with an initial OD_595_ of 0.5, (0.15 for ST4157) were treated with crude extracts of equal concentration of TN72_pal, TN72*_*cpl‐1 or TN72_control. (b) TN72_pal and TN72_control extracts were added to cultures of *S. pneumoniae* (16 NP3, serotype 19F), *Escherichia coli, Staphylococcus aureus* and *Streptococcus pyogenes* (initial OD_600_ 0.8–1.0). The graphs show the decrease in OD per minute (divided by 0.001) in the linear range with changes in the OD of the corresponding control subtracted from the results. ST65 = serotype 6A, ST176 = serotype 6B, ST1390 = serotype 6C, ST4157 = serotype 6B, 35 NP1. The error bars show ± one standard deviation ((a) *n* = 3, (b) *n* = 2)).

To analyse whether Pal and Cpl‐1 produced in the *C. reinhardtii* chloroplast are biologically active, we performed turbidity reduction assays (TRA) with the target bacterium *Streptococcus pneumoniae*. In a TRA, the lytic activity of an enzyme is measured as a decrease in optical density (OD_595_) of a bacterial suspension. Deoxycholate, which induces rapid autolysis of *S. pneumoniae*, was used as a positive control (Mellroth *et al*., 2012). As seen in Figure 6, after the addition of a crude extract from TN72_pal or TN72_cpl‐1, the OD_595_ of a *S. pneumoniae* suspension decreased in comparison with assays using crude extract from TN72_control or untreated controls. In control assays, the OD_595_ showed a gradual decline, which is most likely caused by autolysis of *S. pneumoniae*. The lytic activity of Pal and Cpl‐1 was confirmed in more than 10 independent TRAs. Crude extracts containing either HA‐tagged or untagged Pal caused comparable rates of lysis and decreased the OD_595_ to a similar extent in five independent TRAs. This suggests that the HA‐tag does not interfere with the enzymatic activity of the endolysin. Furthermore, we found that the extracts containing Pal and Cpl‐1 were active against different clinical isolates of *S. pneumoniae*, with all four serotypes (6A, 6B, 6C and 19F) showing lysis including strain ST4157 that is intermediate resistant to penicillin (MIC (minimal inhibitory concentration): 0.125 μg/mL) and resistant to co‐trimoxazole (MIC: 6 μg/mL) (Figure 7a and S9). The specificity of Pal was demonstrated by performing TRAs using *Escherichia coli, Staphylococcus aureus* and *Streptococcus pyogenes*. As shown in Figure 7b and S10, the algal extract containing Pal, which was active against *S. pneumoniae* cells, did not have a measurable effect on the tested bacteria. This suggests that the algal‐produced Pal has specificity for *S. pneumoniae* as described for bacteria‐produced Pal and other endolysins specific to Gram‐positive bacteria (Loeffler *et al*., 2001; Schmelcher *et al*., 2012).

**Figure 8:**
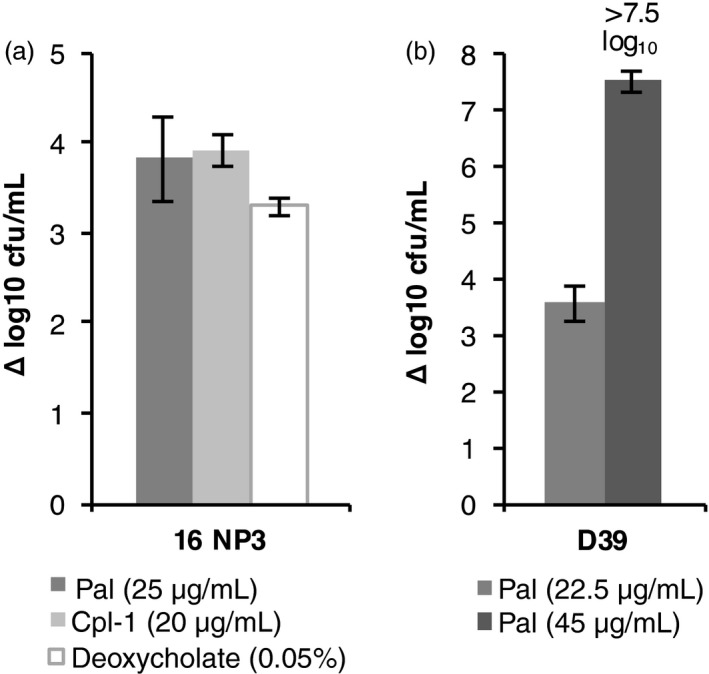
*In vitro* killing of *S. pneumoniae* by isolated Pal and Cpl‐1. (a) *S. pneumoniae* 16 NP3 or (b) D39 was incubated with isolated Pal, Cpl‐1 or deoxycholate as a positive control. In (b), 45 μg/mL of Pal killed all *S. pneumoniae* D39 cells and therefore reduced the cfu/mL by more than 7.5 log_10_ units. The graph shows the decrease in bacterial titres in powers of 10 in comparison with an untreated control. The error bars show ± one standard deviation (*n* = 3).

To demonstrate directly that Pal and Cpl‐1 kill *S. pneumoniae*, we determined the number of colony‐forming units per mL (cfu/mL) remaining in a cell suspension after endolysin treatment (Figure 8). One complication we encountered in initial experiments using the crude algal extracts was the presence of an endogenous bactericidal activity in *C. reinhardtii*, as has been previously reported (Ghasemi *et al*., 2007; Jørgensen, 1962). While this activity did not cause cell lysis (see the TN72_control in Figure 6), it did have a marked effect on the viability of *S. pneumoniae* and therefore masked the effect of the endolysins in the titre assay (data not shown). To demonstrate the killing of *S. pneumoniae* by Pal and Cpl‐1 without the interference of this activity, we repeated the assays with the isolated protein. The addition of 25 μg/mL of Pal or 20 μg/mL Cpl‐1 reduced the bacterial titre of strain 16 NP3 by nearly 4 log_10_ units (Pal: 3.8 ± 0.5 and Cpl‐1: 3.9 ± 0.2 Δlog_10_ cfu/mL) in comparison with an untreated control. Treatment of *S. pneumoniae* D39 with 22.5 μg/mL of Pal reduced the cfu/mL by 3.6 ± 0.3 log_10_ units. A treatment with 45 μg/mL of Pal was sufficient to kill all *S. pneumoniae* cells in suspensions of D39 and 16 NP3, which was a reduction by more than 7 log_10_ units for both strains. This supports the TRA results that the recombinant Pal and Cpl‐1 are able to effectively kill *S. pneumoniae* through cell lysis. Furthermore, it shows that the two endolysins retain antibacterial activity during the purification procedure.

## Discussion

In this study, we investigated the synthesis of two bacteriophage endolysins specific to the human pathogen *S. pneumoniae* in the chloroplast of *C. reinhardtii*. We found that Pal and Cpl‐1 accumulate as predominately full‐length proteins in the transgenic lines. Furthermore, for Pal we demonstrated that this endolysin is highly stable despite the presence of a suite of proteases within the algal chloroplast (Adam *et al*., 2006). The antibacterial activity of Pal and Cpl‐1 against different serotypes of *S. pneumoniae,* including clinical isolates, was demonstrated using both cell lysates and the isolated proteins. Incubation with 25 μg/mL Pal or 20 μg/mL Cpl‐1 resulted in a decrease in colony‐forming units (cfu) for *S. pneumoniae* of nearly four log_10_ units. These activities are similar to the antibacterial activities and minimal inhibitory concentrations (MIC) described for Pal and Cpl‐1 synthesized in *E. coli* or tobacco (Loeffler and Fischetti, 2003; Loeffler *et al*., 2001; Oey *et al*., 2009b; Rodríguez‐Cerrato *et al*., 2007), which suggests that Pal and Cpl‐1 are produced in a predominately active form in the algal chloroplast.

The production of the endolysins did not have any negative impact on the viability of the alga suggesting that the production in *C. reinhardtii* could be performed using continuous cultivation systems. Both proteins account for approximately 1% of total soluble protein (TSP) during mixotrophic cultivation, which equates to ~1.3 mg of recombinant endolysin per gram of cell dry weight. These yields are similar to previous reports on the production of recombinant proteins in the *C. reinhardtii* chloroplast, which range from 0.1% to 5% of TSP (Dreesen *et al*., 2010; He *et al*., 2007; Manuell *et al*., 2007; Rasala *et al*., 2010; Sun *et al*., 2003; Tran *et al*., 2013), although significantly lower than yields reported for tobacco chloroplasts which are typically 10%–30% of the TSP in leaves, and in extreme cases are as much as 70% (Bock, 2015). The reason for this difference is not clear, but might reflect the much tighter anterograde regulation of chloroplast gene expression by the numerous nuclear‐encoded factors in the single chloroplast of the algal cell (Douchi *et al*., 2016; Lefebvre‐Legendre *et al*., 2015). Many of these factors act on the 5′UTR of specific gene transcripts and are required for RNA stability or translation initiation; they therefore also regulate transgenes driven from the same 5′UTR. Competition between endogenous and foreign transcripts with the same 5′UTR for low abundance factors might therefore limit the level of recombinant protein. Indeed, higher yields were achieved using the *psbA* promoter/5′UTR to drive transgene expression in a *psbA* deletion mutant (Manuell *et al*., 2007; Rasala *et al*., 2010). However, the generation of nonphotosynthetic transgenic lines negates the key value of the alga as a light‐driven cell factory; we have therefore focused on maximizing endolysin production in a photosynthetic line. Further studies of the mechanisms of anterograde regulation in the *C. reinhardtii* chloroplast combined with the redesign of UTR elements to eliminate regulatory elements (Specht and Mayfield, 2013) should result in significant improvements in protein production levels (Rasala and Mayfield, 2015).

Microalgae are gaining increasing attention as alternative expression platforms due to the simple and cheap nutrient requirements, as well as easy and cost‐effective scalability, which will potentially result in low production costs for recombinant proteins (Barrera and Mayfield, 2013; Gimpel *et al*., 2014). Furthermore, the GRAS status of several green algal species including *C. reinhardtii*, which lack endotoxins and infectious agents, might decrease downstream processing costs and enables the use of dried algal biomass for the administration of therapeutics and vaccines (Dreesen *et al*., 2010; Gregory *et al*., 2012). However, *C. reinhardtii* cultures reach lower final cell densities and have lower recombinant protein yields than conventional expression systems such as mammalian cells, yeast or bacterial systems (Andersen and Krummen, 2002; Jørgensen *et al*., 2014). Therefore, to determine whether the *C. reinhardtii* chloroplast has the potential to be cost‐competitive with conventional expression platforms, an accurate costing for the production of recombinant proteins at scale in *C. reinhardtii* needs to be generated.

This study represents a first report and ‘proof of concept’ for the production of endolysins in the *C. reinhardtii* chloroplast. The use of endolysins as antibacterial agents is a relatively new, but potentially immense field. It is estimated that, in total, 10^31^ bacteriophages exist globally (Abedon, 2008), so there is a huge untapped resource of endolysins that could be investigated for their potential use as antimicrobials in a wide range of applications (Borysowski *et al*., 2006). A recent study even describes an endolysin that can traverse epithelial membranes and eliminate intracellular *Streptococcus pyogenes* cells (Shen *et al*., 2016). Additionally, several research groups have started to create designer endolysins by removing cell wall binding domains, adding additional catalytic domains or combining domains from different endolysins (Schmelcher *et al*., 2012). Furthermore, endolysins combined with polycationic peptides have been created that are effective against Gram‐negative bacteria. These enzymes are referred to as artilysins (Briers *et al*., 2014). The *C. reinhardtii* chloroplast might offer advantages especially for the production of endolysins and artilysins specific to Gram‐negative bacteria. These enzymes are less specific and can have adverse effects on bacterial production systems containing the peptidoglycan substrate (Briers *et al*., 2014; Oey *et al*., 2009b). The successful production in the *C. reinhardtii* chloroplast of a Gram‐negative endolysin that cannot be produced in bacterial systems would demonstrate a clear advantage of this microalga as an expression platform for endolysins.

Overall it is acknowledged that the field of therapeutic protein production in the green microalga *C. reinhardtii* is in its infancy, and at present is not a viable alternative to more mature technologies. That being said, the use of an algal platform does hold a number of intrinsic advantages over other systems including cost of cultivation and sustainability. The path to greater adoption of this and similar platforms will likely start with niche applications specifically tailored to the unique characteristics exhibited by the algal host; the particular suitability of the *C. reinhardtii* chloroplast for recombinant endolysin expression can be seen as a first step on this road.

## Experimental procedures

### *C. reinhardtii* strains and growth conditions

The recipient strain used to create the transformant lines was TN72, a *psbH* deletion mutant of the cell wall‐deficient *C. reinhardtii* strain cw15.mt+ (Wannathong *et al*., 2016). TN72 was maintained on Tris‐acetate‐phosphate (TAP) medium at 20 °C and 5–10 μmol/m^2^/s light, as were transformant lines following selection on high salt minimal (HSM) medium (Harris, 2009). Liquid cultures were grown in Erlenmeyer flasks at 100–200 μmol/m^2^/s, 120 rpm shaking and 25 °C in either illuminated shaking incubators or Algem photobioreactors (Algenuity, Stewartby, UK) for growth studies. Growth was measured by recording the optical density at 750 nm with a Unicam UV/Vis Spectrometer (Thermo Electron Corporation, USA) or 740 nm in the Algem photobioreactor.

### Bacterial strains and growth conditions

Activity assays were performed with the *S. pneumoniae* strain 16 NP3 (serotype 19F) or D39. Additional assays were with clinical isolates: ST65 = serotype 6A, H08212 0259; ST176 = serotype 6B, H08052 0052; ST1390 = serotype 6C, H05252 0075; ST4157 = serotype 6B, 35 NP1. The *S. pneumoniae* strains and *S. pyogenes* (ATCC 19615, *Streptococcus* group A) were grown on Columbia blood agar plates overnight at 35 °C under anaerobic or aerobic conditions, respectively. Liquid cultures were grown in trypticase soy broth (30 g/L trypticase soy broth, 3 g/L yeast extract) overnight at 35 °C without shaking. Anaerobic conditions were generated using Oxoid AGS CO_2_Gen Compact gas packs and an anaerobic jar from Oxoid. *S. aureus* (ATCC 28213) liquid cultures were grown in ISO‐Sensitest broth from Oxoid overnight at 35 °C with shaking. All target bacteria were obtained from the Royal Free Hospital (London, UK).

### Plasmid construction

Endolysin coding sequences (including a C‐terminal human influenza haemagglutinin (HA) tag sequence) were codon‐optimized for the *C. reinhardtii* chloroplast to a Codon Adaption Index (CAI) of 0.8 using the Kazusa CAI table (Nakamura *et al*., 2000) and synthesized *de novo* by GENEART (Regensburg, Germany). A 5′ SapI and a 3′ SphI restriction site were included in each synthetic gene to facilitate cloning into the chloroplast expression vectors pASapI (*atpA* promoter/5′UTR used to drive GOI expression) and pSRSapI (*psaA* exon 1 promoter/5′UTR) as described in Wannathong *et al*. (2016). Plasmids were prepared from *E. coli* DH5α by alkaline lysis (Sambrook and Russell, 2001) and checked by SphI digestion and by Sanger DNA sequencing (Wolfson Institute for Biomedical Research, University College London). For algal transformation, larger plasmid preparations were made using a QIAfilter Plasmid Midi kit (Qiagen).

### Transformation of *C. reinhardtii*

The endolysin genes were introduced into the *psbH–trnE2* intergenic region of the chloroplast genome of TN72 by homologous recombination, restoring a functional *psbH* gene and the ability to grow photosynthetically. The transformation procedure was based on that described by Kindle (1990). Cells were grown to mid‐log phase (1–2 × 10^6^ cells/mL) in TAP medium, harvested by centrifugation (8000 ***g***, 8 min) and resuspended in TAP medium to 2 × 10^8^ cells/mL. The cell suspension (300 μL per transformation) was vortexed for 15 s together with 0.3 g of glass beads (Sigma‐Aldrich, diameter 0.4–0.6 mm) and 2–20 μg of circular plasmid DNA. Molten top agar (3.5 mL HSM + 0.5% agar) at 42 °C was mixed with the cell suspensions and poured onto HSM plates. The plates were incubated for 1 h in the dark, followed by 3–4 weeks at 20 °C under 50 μmol/m^2^/s light. To confirm gene insertion and homoplasmicity of transformants*,* DNA was extracted using the Chelex 100 method as described by Werner and Mergenhagen (1998), and 2 μL of the DNA extract used in PCR reactions with the primers F1, R1 and R3 (Young and Purton, 2014).

### Southern blot analysis

Genomic DNA (4 μg) of the recipient strain TN72, TN72_pal and TN72_cpl‐1 was cut with the restriction enzymes SphI and EcoRI and separated on an agarose gel (1% w/v). The DNA was transferred to a Hybond N (Amersham Biosciences) membrane as described in Sambrook and Russell (2001). Subsequently, the blot was hybridized with DNA probes (produced either by PCR or the digest of a plasmid) that bind within the *pal* and *cpl‐1* genes, as well as a probe that binds just before the insertion site of the expression cassette in all strains. DNA labelling and detection was performed with the DIG High Prime DNA Labelling and Detection Starter Kit II (Roche Diagnostics GmbH) according to the manufacturer's instructions.

### SDS‐PAGE and Western blot analysis

SDS‐PAGE was carried out using the Mini‐PROTEAN Tetra cell system (Bio‐Rad) and gels based on the recipe by Laemmli (1970) containing 15% acrylamide. Samples were supplemented with sample loading buffer, boiled at 99 °C for 3 min and centrifuged at 21 000 ***g*** for 2 min. The gels were run at 120–150 V for 90–120 min. To visualize all proteins, the gels were stained with Coomassie Brilliant Blue R solution for 1 h and destained for at least 2 h. The stained gels were scanned using the Odyssey^®^ imaging system from LI‐COR.

For Western blot analyses, the proteins were transferred to Hybond‐ECL nitrocellulose membranes (GE Healthcare), at 19 V for 1 h using a Trans‐Blot SD semi‐dry electrophoretic transfer cell (Bio‐Rad). The membranes were blocked overnight in TBS‐T (TBS + 0.1% Tween) with 0.5% milk and incubated with the primary antibody (α‐HA antibody from rabbit (Sigma‐Aldrich product H6908) diluted 1:2000 or anti‐Pal antibodies) for 1–3 h followed by incubation with the secondary antibody for 1 h. Both antibodies were resuspended in TBS‐T + 0.5% milk, and the membranes were washed after each incubation 3× in TBS‐T for 5–10 min. When ECL IgG horseradish peroxidase‐linked secondary antibodies (GE Healthcare, 1:10 000) were used, the membranes were incubated with SuperSignal^®^ West Pico Chemiluminescence Substrate (Thermo Scientific) for 5 min and exposed to Hyperfilm ECL (GE Healthcare). For quantitative Western blot analyses, IRDye^®^ secondary antibodies (Dylight™ 800, Thermo Scientific, 1:20 000) and the Odyssey^®^ Infrared Imaging system (Li‐COR Biosciences) were used. Custom made anti‐Pal antibodies were raised by Eurogentec (Belgium) using two peptides of 17 amino acids (see Figure S2) attached to keyhole limpet haemocyanin carrier protein.

### Preparation of *C. reinhardtii* extract

*Chlamydomonas reinhardtii* cultures were grown to an OD_750_ of 2–3, harvested by centrifugation at 8000 *g* for 10 min and resuspended in 20 mm sodium phosphate (Na‐Pi) buffer (pH 6.9) + protease inhibitor (Roche cOmplete, EDTA‐free). Cells were either broken by three cycles of freezing and thawing (liquid nitrogen, 30 °C) or sonication with the Cup Horn (Qsonica, LLC), followed by centrifugation at 21 000 ***g*** for 5 min (=crude extract).

### Endolysin isolation and quantification

After cell breakage, cell suspensions were centrifuged at 5000 ***g*** for 20 min, followed by ultracentrifugation at 100 000 ***g*** for 1 h. Ultracentrifugation extracts were applied to a diethylethanolamine (DEAE) cellulose column. The column was washed with six column volumes (CV) of Na–Pi, eight CV of Na–Pi + 1.5 m NaCl and four CV of Na–Pi + 0.1 m NaCl. Pal and Cpl‐1 were eluted with 2 CV of Na–Pi + 0.1 m NaCl + 6.5% (w/v) choline. The elution fractions with the highest amount of Pal and Cpl‐1 were determined in protein microarrays (2 μL spots on nitrocellulose membranes, followed by Western blot analysis protocol), combined and dialysed using Slide‐A‐Lyzer G2 dialysis cassettes (20 000 MWCO) (Thermo Scientific). After dialysis, the elution was concentrated 10‐fold by ammonium sulphate (AS) precipitation: Pal was precipitated with 35% AS, and Cpl‐1 with 50% AS. Alternatively, the chromatography as described above was performed with an ÄKTA pure system (GE Healthcare Life Sciences) and the fractions with the highest amount of endolysin were concentrated with centrifugal concentrators (5000 MWCO). Protein concentrations were determined using Bradford reagent (Sigma‐Aldrich) and bovine serum albumin as standard (taking impurities into account).

Endolysin concentration within *C. reinhardtii* lines TN72_pal and TN72*_*cpl‐1 was quantified using serial dilutions of the isolated Pal or Cpl‐1 in Western blot analyses with anti‐HA antibodies and IRDye^®^ secondary antibodies. The IR fluorescence signals of the serial dilutions were plotted against the protein concentration (determined by Bradford assay, taking impurities into account), and the equations of the resulting trend lines were used to calculate the amount of Pal/Cpl‐1 in cell lysates. The amount of total soluble protein (TSP) in the cell suspensions was determined by Bradford assay after cell breakage and centrifugation at 13 000 ***g*** for 15 min (Manuell *et al*., 2007). The cell dry weight was determined after lyophilization of the cell suspensions for 16 h.

### Assays of endolysin activity

*S. pneumoniae* cultures were grown to mid‐log phase, (*S. pyogenes, E. coli* and *S. aureus* overnight) harvested and resuspended in 20 mm Na–Pi. Most TRAs were performed at 37 °C in 96‐well microtitre plates with 20 μL of crude extract in a final volume of 200 μL using a FLUOstar OPTIMA Microplate Reader (BMG Labtech Ltd) recording the OD_595_ over times courses of 60–180 min. The assays in Figure 7b and S10 were performed in cuvettes with 50 μL of crude extract in a final volume of 1 mL at room temperature. For the preparation of the crude extracts used in the TRAs, cultures of TN72_pal, TN72_cpl‐1 and TN72_control were grown to the same OD_750_, harvested by centrifugation and resuspended in 1/100 of the culture volume. After cell breakage, the suspension was centrifuged at 21 000 ***g*** for 5 min and the supernatant was used as crude extract in the TRAs.

To determine the colony‐forming units (cfu) that survived the endolysin treatment, samples from TRAs with the isolated endolysins were diluted in serial 10‐fold dilutions and plated in triplicate onto Columbia blood agar plates. After incubation overnight, the cfu were counted and the decrease in cfu/mL calculated relative to an untreated control. For the cfu assays with *S. pneumoniae* D39, frozen aliquots (–80 °C) from an overnight culture were thawed and incubated with isolated endolysin at 37 °C for up to 200 min. The decrease in cfu was determined as described above.

## Supporting information


**Figure S1** The *pal* and *cpl‐1* sequences codon‐optimised for the *C. reinhardtii* chloroplast.
**Figure S2** Southern blot analysis confirming the predicted insertion of *pal* and *cpl‐1* into the plastome of TN72.
**Figure S3** Protein sequence of Pal showing the peptide sequences used to raise antibodies.
**Figure S4** Growth of *C. reinhardtii* TN72_pal compared to the TN72_control.
**Figure S5** Comparison of the amount of Pal produced using the *atpA* or *psaA* exon 1 promoter/5′UTR elements.
**Figure S6** Comparison of the amount of Cpl‐1 produced using the *atpA* or *psaA* exon 1 promoter/5′UTR elements.
**Figure S7** Yield of Pal at different growth stages and the influence of darkness on the protein stability during stationary phase.
**Figure S8** Yield of Pal per culture volume at different growth stages during mixotrophic and heterotrophic growth.
**Figure S9** Turbidity reduction assay showing the lytic activity of crude cell extracts containing Pal and Cpl‐1 against clinical isolates of *S. pneumoniae*.
**Figure S10** Turbidity reduction assays showing the specific lytic activity of Pal produced in *C. reinhardtii* against *Streptococcus pneumoniae* compared to *Escherichia coli*,* Streptococcus pyogenes* and *Staphylococcus aureus*.
