# Supplementary material for: Synthesis of bacteriophage lytic proteins against Streptococcus pneumoniae in the chloroplast of Chlamydomonas reinhardtii
Source: Plant Biotechnol J. 2017 Mar 7;15(9):1130–40. doi: 10.1111/pbi.12703 (PMC5552482; doi:10.1111/pbi.12703)
Supplement: Supplementary file 1 — Figure S1 The pal and cpl‐1 sequences codon‐optimised for the C. reinhardtii chloroplast. Figure S2 Southern blot analysis confirming the predicted insertion of pal and cpl‐1 into the plastome of TN72. Figure S3 Protein sequence of Pal showing the peptide sequences used to raise antibodies. Figure S4 Growth of C. reinhardtii TN72_pal compared to the TN72_control. Figure S5 Comparison of the amount of Pal produced using the atpA or psaA exon 1 promoter/5′UTR elements. Figure S6 Comparison of the amount of Cpl‐1 produced using the atpA or psaA exon 1 promoter/5′UTR elements. Figure S7 Yield of Pal at different growth stages and the influence of darkness on the protein stability during stationary phase. Figure S8 Yield of Pal per culture volume at different growth stages during mixotrophic and heterotrophic growth. Figure S9 Turbidity reduction assay showing the lytic activity of crude cell extracts containing Pal and Cpl‐1 against clinical isolates of S. pneumoniae. Figure S10 Turbidity reduction assays showing the specific lytic activity of Pal produced in C. reinhardtii against Streptococcus pneumoniae compared to Escherichia coli, Streptococcus pyogenes and Staphylococcus aureus. [file PBI-15-1130-s001.docx]

| **** |
| --- |
| **Figure S1.** The *pal* and *cpl-1* sequences codon-optimised for the *C. reinhardtii* chloroplast to a Codon Adaption Index (CAI) of 0.8 using the Kazusa CAI table (Nakamura et al. 2000). Start and Stop codons are highlighted in red, the C-terminal human influenza haemagglutinin (HA) epitope tag in blue. |

| **** | |  |  |
| --- | --- | --- | --- |
| **** | | | |
|  | **Figure S2.** Southern blot analysis confirming the predicted insertion of *pal* and *cpl-1* into the plastome of TN72. Genomic DNA of the recipient strain TN72, TN72_pal and TN72_cpl-1 was cut with SphI and EcoRI and separared on an agarose gel. After transferring the DNA to a Hybond N membrane, the blot was hybridized with probes binding within the *pal* and *cpl-1* genes, as well as a probe that binds adjacent to the insertion site of the expression cassette in all strains. (a) shows the restriction sites, probe binding sites and the expected band sizes, (b) the Southern blot. |  |  |

| **** |
| --- |
| **Figure S3.** Protein sequence of Pal showing the peptide sequences used to raise antibodies highlighted in red and blue. The two synthesized peptides have a predicted high immunogenicity and were used to raise antibodies in rabbits by the company Eurogentec (Belgium). A cysteine was added to the *N* terminus of peptide 1 to target the coupling site on the keyhole limpet hemocyanin (KLH) carrier protein. |

| **** |
| --- |
| **Figure S4.** Growth of *C. reinhardtii* TN72_pal compared to TN72_control. TN72*_*pal and TN72_control were grown under continuous shaking at 120 rpm, 25 ºC and with a light intensity of 200 μmol/m^2^/s in Tris-acetate-phosphate (TAP) medium in the Algem photobioreactor (Algenuity, Stewartby, UK). The OD_740_ was automatically recorded every 0.5 h and the biomass (cell dry weight) was determined at four time points during growth. The error bars show ± one standard deviation (n = 2).  **** |
|  |
| **Figure S5.** Comparison of the amount of Pal produced using the *atpA* or *psaA* exon 1 promoter/5’UTR elements. Cultures of TN72_pal with *pal* under the control of the *psaA* exon 1 and *atpA* promoter/5’UTR were grown in duplicate under standard conditions and samples were taken at multiple time points during growth, concentrated five times in Na-Pi-buffer and 20 μl were analysed in a western blot analysis with anti-HA antibodies, IRDye® secondary antibodies and the Odyssey® Infrared Imaging System for detection (a) and signal quantification. The IR fluorescence signals were divided by the optical density (750 nm) of the culture at the time of sampling and are shown in (b). The error bars show ± one standard deviation (n = 2). |

| **** |
| --- |
| **Figure S6.** Comparison of the amount of Cpl-1 produced using the *atpA* and *psaA* exon 1 promoter/5’UTR elements. Cultures of TN72_cpl-1 with *cpl-1* under the control of the *psaA* exon 1 and *atpA* promoter/5’UTR were grown under standard conditions. Samples were diluted to 1.0x, 0.2x and 0.1x the culture volume and 20 μl were analysed by western blot with anti-HA antibodies, IRDye® secondary antibodies and the Odyssey® Infrared Imaging System for detection and quantification. |

| **** |
| --- |
| **Figure S7.** Yield of Pal at different growth stages and the influence of darkness on the protein stability during stationary phase. Cultures of TN72_pal were grown under standard conditions and samples were taken at multiple time points during growth. After harvesting the samples were normalized to the optical density (750 nm) at the time of sampling. The samples were analysed in western blot analyses with anti-HA antibodies, IRDye^®^ secondary antibodies and the Odyssey^®^ Infrared Imaging System for detection and quantification. All cultures were grown in the light (200 µmol/m^2^/s) at the beginning of the experiment. Then one half was moved to the dark after 58 h (dark blue) and the other half remained in the light (light blue). (a) shows one of the western blot images; (b) the averages of the Odyssey IR fluorescence signals, and (c) the growth of the cultures. The error bars in (b) show ± one standard deviation (n = 2). |

| **** |
| --- |
| **Figure S8.** Yield of Pal per culture volume at different growth stages during mixotrophic and heterotrophic growth**.** Cultures of TN72_pal were grown with acetate under mixotrophic conditions in the light (light blue) and heterotrophic conditions in the dark (dark blue). Samples were taken at multiple time points during growth, after harvest all samples were resuspended in an equal volume (a five times concentration of the culture volume). The samples were analysed in western blot analyses with anti-HA antibodies, IRDye^®^ secondary antibodies and the Odyssey^®^ Infrared Imaging System. (a) shows one of the western blot pictures; (b) shows the average of the Odyssey IR fluorescence signals and (c) the growth curves of the cultures The error bars in (b) show ± one standard deviation (n = 4, two cultures, each analysed in duplicates), the error bars in (c) show the standard deviation between the two cultures (n = 2). L = Culture grown mixotrophically in the light, D = Culture grown heterotrophically in the dark.   \|  \| \| --- \| \| **Figure S9.** Turbidity reduction assay showing the lytic activity of crude cell extracts containing Pal and Cpl-1 against clinical isolates of *S. pneumoniae.* Bacterial suspensions with an initial OD_595_ of 0.5, (0.15 for ST4157) were treated with crude extract of equal concentration of TN72_pal, TN72*_*cpl-1 or TN72_control and the OD595nm was measured over a time course at 37ºC. (a) ST65 = serotype 6A, H08212 0259; (b) ST176 = serotype 6B, H08052 0052; (c) ST1390 = serotype 6C, H05252 0075; (d) ST4157 = serotype 6B, 35 NP1. The error bars show ± one standard deviation (n = 3). \|  \| **** \| \| --- \| \| **Figure S10.** Turbidity reduction assays showing the specific lytic activity of Pal produced in *C. reinhardtii* against *Streptococcus pneumoniae* (a) compared to *Escherichia coli* (b), *Streptococcus pyogenes* (c) and *Staphylococcus aureus* (d). Bacterial suspensions with an initial OD_600_ of 0.8. to 1.0 were treated with crude extract of equal concentration of TN72_pal or TN72_control and the OD600nm was measured over a time course at room temperature. *S. aureus* cells had been heat inactivated at 80ºC for 10 min before the assay, the other species were used alive just after harvest. The error bars show ± one standard deviation (n = 2). The error bars show ± one standard deviation (n = 2). \| |
